# Why the Elective Caesarean Lottery is Ethically Impermissible

**DOI:** 10.1007/s10728-019-00370-0

**Published:** 2019-04-29

**Authors:** Elizabeth Chloe Romanis

**Affiliations:** Centre for Social Ethics and Policy, School of Law, University of Manchester, Williamson Building, Oxford Road, Manchester, M13 9PL UK

**Keywords:** Autonomy, Caesarean section, Elective caesareans, Pregnant women, Childbirth, Birth ethics

## Abstract

In the United Kingdom the law and medical guidance is supportive of women making choices in childbirth. NICE guidelines are explicit that a competent woman’s informed request for MRCS (elective caesarean in the absence of any clinical indications) should be respected. However, in reality pregnant women are routinely denied MRCS. In this paper I consider whether there is sufficient justification for restricting MRCS. The physical and emotive significance of childbirth as an event in a woman’s life cannot be understated. It is, therefore, concerning that women are having their wishes ignored, and we must ascertain whether the denial of agency is justifiable. To answer this question I first demonstrate that access to MRCS is a lottery in the UK. Second, I argue that there is nothing unique about pregnancy that displaces the ethical norm of respecting patents’ sufficiently autonomous choices. Thus, the starting presumption is that all informed choices regarding MRCS should be respected. To ascertain whether any restriction of MRCS is justifiable the burden of proof must be placed on those who argue that MRCS is ethically impermissible. I argue that the most common justifications in the literature against MRCS are insufficient to displace the presumption in favour of autonomous choice in childbirth. I conclude that MRCS should be available to pregnant women, and we must strive to reduce the lottery in access to choice.

## Introduction

UK law and medical guidelines support women having access to choices in childbirth, including *maternal request caesarean section* (MRCS). The reality is, however, that women are routinely denied MRCS [9]. This paper focuses on whether there is a sufficient justification for denying women this option. In medicine the prevailing ethical norm is respect for patient autonomy. In this paper I argue, therefore, that the burden of proof in the MRCS debate must be shifted onto those advocating that MRCS is unethical. If women are to be denied choice in childbirth, opponents of MRCS must prove that it is an exception to predominant ethical norms. In support of MRCS provision I demonstrate that the reasons advanced for restricting MRCS in the literature are not sufficient to rebut the presumption in favour of sufficiently autonomous choices.

Caesareans^1^ were initially designed, and made increasingly available with advancing surgical technology,^2^ for emergencies to save the lives of pregnant woman and foetuses. Caesareans are life saving when vaginal birth poses a risk to pregnant woman or foetus, for example where the foetus is distressed [59] or the pregnant woman develops a serious health condition [59]. It was only as caesareans became more routine [12] in the mid-1980s [63] that ‘elective caesarean’ emerged as a concept [67]. Caesareans are now the most common surgical procedure performed worldwide [16] and are safer than ever before [57]. In England 29% of births are by caesarean [42]. At least some of these caesareans are believed to be MRCS.^3^ These are planned caesareans in low-risk pregnancies in the absence of any (maternal or foetal) clinical indications at the pregnant woman’s request [61]. Women cite lots of reasons for choosing MRCS including tokophobia [63],^4^ previous trauma [9], to avoid pelvic floor injury [43], or convenience [6]. The National Institute of Health and Care Excellence (NICE) guidelines advise doctors that MRCS is permissible when women make an informed choice with capacity [40]. Moreover, English law is clear, following *Montgomery v Lanarkshire* [39], that doctors must discuss ‘all reasonable alternatives’ with pregnant patients including choices about delivery. In theory, women are empowered to make choices about childbirth. Birthrights, a UK charity advocating for the rights of women in childbirth, concluded in their 2018 report, however, that pregnant women often cannot access MRCS [9]. Many NHS Trusts explicitly or implicitly prohibit MRCS [9], and there is inconsistent interpretation of the NICE guidelines [9]. Pregnant women are routinely denied choices in childbirth and this is often the result of a belief that MRCS is ethically impermissible. In this paper I first demonstrate the extent to which access to MRCS in the UK is a lottery. I highlight the important findings of the Birthrights report; guidelines about MRCS are being ignored, misinterpreted or inconsistently applied by different NHS trusts. Moreover, I uniquely demonstrate how MRCS is often inaccessible because of the pervasive influence of doctor’s opinions, and societal influences, over women’s childbirth choices. Finally, I will consider the common arguments advanced against MRCS arguing that none are sufficient justification to prevent women making free choices about childbirth.

Childbirth is often characterised as an important issue of reproductive autonomy; because how a pregnancy is delivered is a reproductive choice [12]. This conceptualisation negates the substance of a choice about childbirth. A woman’s choice of delivery is not about reproduction, but how she uses her *body* to complete reproduction. MRCS is about a woman’s body, her bodily autonomy and integrity. This paper argues all women are entitled to decide what physical consequences of reproduction they are willing to assume. The consequences of allowing some women choice and not others, in a lottery, can be devastating for those denied agency. Women in the UK are concerned about their choices in childbirth. The most common reason Birthrights are contacted is for clarification about access to or assistance accessing MRCS [9]. Where pregnant women live, the policies of local Trusts and the opinions of obstetricians attending to them are determinative of the choice women have. The significance of ‘giving birth’ as an event in a woman’s life is obvious, and evidence suggesting that women are having their wishes ignored is of serious concern. Unsurprisingly, MRCS access has captured significant public attention in the UK [58], and prompted discussion of the ethical permissibility of MRCS [53]. The public scrutiny that such a personal aspect of a pregnant woman’s life has been subjected to in 2018 is yet another manifestation of how little trust practitioners place in women making decisions about pregnancy. The importance of examining the permissibility of and access to MRCS cannot be overstated.

## MRCS: The Current Lottery

NICE guidelines concerning MRCS were amended in 2011. They state that MRCS should not be routinely offered, but doctors should perform MRCS when they are confident a woman is making an informed decision and vaginal delivery (VD) is unacceptable to her [40]. The guidelines stipulate that her reasons for the request and the risks and benefits of different childbirths must be thoroughly discussed [40]. Further, if a woman’s request results from fear about VD she should be offered expert mental health support [40]. If an obstetrician is unwilling to perform MRCS at the request of an informed patient she should refer the patient to another colleague to perform the procedure [40]. The guidelines' aims are explicit: to ensure that the views of pregnant women are central in decisions about childbirth, and that pregnant women are empowered to make informed decisions [40]. However, the choice the guidelines try to protect is often not available in reality.

A doctor cannot be legally compelled to comply with a request for elective caesarean. The case of *R v GMC* (*ex parte Burke*) clarified both that a patient has no legal right to demand one form of treatment over another, and that a doctor cannot be compelled to perform any particular medical intervention requested by a patient in the absence of emergency [49]. Moreover, whilst it is good medical practice to follow NICE guidelines there is no legal requirement to do so. The NICE Charter makes explicit provision for doctors to depart from guidelines.^5^ It could be the case, however, that MRCS features in the medical professional’s duty to disclose following *Montgomery v Lanarkshire* [39] and *Birch v University College Hospitals NHS Trust* [8]. MRCS might form a part of the duty to disclose reasonable options emerging from these cases, if MRCS and VD are comparatively risky (as I argue is the case later in this paper). *Birch* [8], in particular, potentially legally substantiates the NICE guidelines [40], as it was the first case in English law to find that there is a duty to disclose feasible alternative treatment options [27]. Regardless of whether MRCS features in the duty to disclose, the guidelines and the law are clear that doctors cannot be compelled, outside of emergencies, to perform treatments they are uncomfortable with.^6^ Thus, doctors having a duty to disclose would require them to mention  MRCS to patients, but they would subsequently remain able to refuse to perform the procedure if it was requested. The availability of MRCS is, thus, dependent upon individual doctors being willing to perform the procedure, and of Trusts adopting policies allowing doctors to perform the procedure. Birthrights highlighted there is an inconsistent approach to implementing NICE guidelines across NHS trusts. 15% of NHS trusts explicitly do not offer MRCS. 47% either effectively did not offer MRCS or only partially offered MRCS [9]. Within this 47% there was uncertainty about policy or inconsistency in application. Many Trusts had no written policy on MRCS. Birthrights argue this indicates thoughtlessness and inconsistency [9]. Lack of written guidance makes it harder for women to know what their choices are, and easier for doctors to evade following the NICE recommendations. Other Trusts had incomplete guidelines or policies with significant barriers to access such as requiring the permission of multiple doctors or restrictions [9]. Birthrights also highlight that availability of MRCS is further restricted by local Clinical Commissioning Groups' (CCGs) decisions not to fund them. 13% of CCGs were explicitly unsupportive of MRCS, but there is reason to believe this is a conservative estimate [9]. MRCS is often categorised as of ‘low-clinical value’ and only funded in limited circumstances [9]. It is routinely deprioritised by CCGs, therefore Trusts have to adopt restrictive policies. CCGs and Trust policies combined effectively prevent some (and we do not know how many) women making choices about childbirth.

Even within Trusts allowing MRCS, the NICE guidelines still place a considerable amount of power in doctors’ hands because their views are prioritised over women’s choices. A doctor’s right to refuse to perform MRCS^7^ effectively contradicts the stated aim of the guidelines to make women primary decision-makers. It is only if women can convince a medical practitioner they should be allowed MRCS that they are empowered to make a genuine choice about childbirth. Doctors' personal opinions about MRCS will affect the access their patients are afforded. Surveys indicate different levels of willingness amongst UK obstetricians to perform MRCS. In 1997 30% of British obstetricians indicated they would perform MRCS [2]. A more recent study places the estimate closer to 70% [18]. MRCS is gaining credence amongst British doctors,^8^ but 30% likely to refuse is still significant enough to generate concern about women’s access to choice. There are interesting parallels between abortion legislation allowing conscientious objection [1], and NICE guidelines about MRCS. The critiques of the medical gate-keeping of abortion [56] therefore also apply in the MRCS context. However, the number of doctors unwilling to perform MRCS far outstrips the number of doctors unwilling to perform abortion.^9^ This amplifies the criticism that can be levied against the medical profession’s grip on decisions about pregnancies. Conscientious objection, I argue, is inappropriate in this context.^10^ If a doctor feels that they could never be comfortable with performing MRCS, it would be prudent of them to conscientiously object by electing to enter a medical speciality other than obstetrics and gynaecology [55], rather than by refusing to respect any individual woman’s choice about childbirth.

In the absence of guidance for MRCS counselling the advice given is likely to be personal and significantly affected by the doctor’s personal and clinical experiences [7]. The evidence about risks and benefits in MRCS is often inconclusive.^11^ It would be easy for well-meaning clinicians to emphasise certain concerns over others, or to weight the significance of particular risks above others. It is difficult for patients to challenge the opinions of doctors, or to judge whether the advice they receive is based on a fair interpretation of medical evidence. Moreover, the inherent power dynamic [29] in the doctor–patient relationship might result in undue pressure to make certain choices. This is a ‘particular challenge for women socially conditioned to be “compliant”’ [10]. Some women may be being unfairly denied opportunities to make true MRCS choices because their medical counsel is, even unintentionally, unduly influencing them. This will be most evident in those cases where the NICE guidelines or Trusts policies indicate that counselling, because of their choice, is necessary [40]. Further, even amongst doctors that indicated a willingness to perform MRCS, data suggests that the reason for a request will influence their decision to perform MRCS [64]. NICE guidelines explicitly encourage doctors to evaluate *why* the patient seeks MRCS before making a decision [40]. This effectively creates a ‘rationality’ standard for decision-making (at odds with English law).^12^ The guidelines imply a hierarchy in reasons for MRCS that might indicate different responses. This disadvantages women with ‘less socially acceptable’ reasons, or women who feel uncomfortable divulging their reasons^13^ who may be denied MRCS because their reason(s) are *notgood enough*. Fundamentally, it is unfair to subject women to this level of scrutiny in reasoning in a decision about their body, rather than just ensuring they understand the risks involved in their choice. Rational decisions are not required for other aspects of medical decision-making even when decisions are considered clinically unwise [51]. Examining MRCS reasoning treats women differently by virtue of pregnancy. Finally, the requirement for doctors unwilling to perform MRCS to refer patients making informed requests [40] is no guarantee of MRCS access. If the Trust has an explicit policy against MRCS a pregnant woman would have to be referred to a different Trust. This care may be inaccessible to her because of distance or cost. There could also be pregnant women who receive unclear counselling that suggests they cannot seek advice elsewhere, or that another doctor would also refuse MRCS.

There is no reliable data about what percentage of caesareans performed are MRCS [46]. While some data about elective caesareans in England is collected,^14^ there is no data about how many requests are denied. It is clear, however, that these barriers combined have resulted in a lottery accessing choice in childbirth. Transparency ‘is long overdue’ [53]. Inconsistent access to choice is always detrimental. If there were sound ethical reasons to limit MRCS we need to establish what these ethical reasons are and when limitations in choice should be applied. Restrictions, if justified, should be consistent  in order to prevent uncertainty about when, where and how MRCS happens. If women, as advocated by the NICE guidelines, should be entitled to choose their childbirth decisions made by organisations and individual doctors that prevent choice are harmful. The existence of a lottery in choice, moreover, is likely to impact most on women already disenfranchised. At present only women with the means to seek out information about MRCS, with education that empowers them to understand what they can demand from doctors, and the means to seek assistance elsewhere if necessary are able to *ensure* access to choice.

## Is MRCS Ethically Impermissible?

Patient autonomy and an individual’s right to bodily integrity are usually seen as paramount in the context of medical decision-making. This is because autonomy is an indispensable component of individual wellbeing. Respecting maximally autonomous choices affords respect to individual’s preferences and values [37], thus allowing individuals to pursue their conception of the good life [14]. Individual liberty, though important to distinguish from autonomy [17] is also crucial to the ability of individuals to act according to their own value-systems and commitments. The ability to control one’s own body is crucial; a prerequisite to freedom of action, without which individuals are deprived of the ability to make free choices. Enabling individuals the freedom to determine what happens to their own body enables individuals to make an autonomous decision, which facilitates discreteness of self [35], enables them to take responsibility for decisions [19] and maintains self-realisation [35]. This is because when making decisions individuals can critically reflect on their own subjective preferences. We can only assess a person’s autonomous decisions by reference to his or her own value system. The rights of any person are not then diminished by pregnancy. In fact, pregnancy is a uniquely personal period in a woman’s life [10] that can be incredibly emotive. Childbirth is a difficult and life-changing (potentially life-ending) process. Issues of agency are, thus, crucial in ensuring empowerment and ownership of pregnancy and the difficult role imposed on women by sexual reproduction. Denying women choices about childbirth forces them to relinquish their identity by surrendering to certain life-altering experiences over others. Promoting, or only allowing, one form of birth prevents the focus of pregnancy being the complex and personal needs of any individual pregnant woman [53]. For these reasons we should respect women’s choices in childbirth. The law governing medical procedures strongly supports individual decision-making [51] during pregnancy [50]. Autonomy is not the only important factor in determining the ethical permissibility of medical procedures [30]; however overriding sufficiently autonomous choices is an exceptional way to treat patients.^15^ Restricting a sufficiently autonomous preference for MRCS can only be justified if it can be demonstrated that MRCS is an extraordinary procedure and there are, therefore, reasons to restrict access. In this section I demonstrate that most of the arguments attempting to make such a claim do not stand up to scrutiny. Thus, there is not sufficient justification to prevent pregnant women making meaningful decisions about their body.

The arguments usually made against MRCS fit neatly into the four principles framework of bioethical reasoning advocated by Beauchamp and Childress [5]. Categorising the arguments in this way helps bring more order to the debate, and to understand if any of these arguments is sufficient justification for the restriction of maximally autonomous MRCS. First, I consider arguments of beneficence based on the risks of MRCS and whether women can and should be able to assume these risks. Second, I consider arguments of non-maleficence based on MRCS being an unnecessary intervention. Finally, I consider arguments of justice based on claims that MRCS is an inequitable allocation of resources.

## Beneficence: Additional (?) Risk

Most objectors to MRCS are concerned with the additional risks of caesarean, ranging from increased risk of maternal mortality to increased risk of infection or haemorrhage [41] and respiratory distress in neonates [46]. MRCS also carries the additional and inherent risks of surgery such as use of anaesthetics [46]. Critics of MRCS argue that MRCS allows pregnant women to expose themselves to potential additional harms [11] and it is in pregnant women’s best interests to refrain from these unnecessary caesareans that create additional risks to her health and her foetus. This argument has several premises. First, women cannot make maximally autonomous choices about MRCS. Second, women (even if capable of making an autonomous choice) should not be able to choose to assume additional unnecessary risks in childbirth. Finally, that MRCS harbours unjustifiable additional risks.

## Prejudice Against Autonomy in Childbirth

The idea that doctors can unilaterally exercise therapeutic privilege to make decisions for their patients has long been rejected in medical ethics and law.^16^ The claim that women should not, or cannot, choose MRCS because of increased risk, however, demonstrates that paternalistic thinking has, inconsistent with the broader approach in healthcare [28], continued to flourish in the childbirth context. A perpetuated myth that pregnancy is ‘different’ potentially encourages doctors to substitute women’s choices for best interests judgements. It is sometimes argued that pregnant women, on agreeing to carry a pregnancy to term,^17^ assume some responsibilities towards the foetus [48]: potentially including choosing the least risky childbirth. MRCS does not necessarily involve women making riskier choices for foetuses.^18^ Regardless, denying women choice about childbirth reduces women to ‘foetal incubators’ and denies individual agency. Childbirth is a physically demanding process irrespective of mode of delivery. Denying women choice forces one childbirth rather than another, treats women as a mere means [47] to ‘deliver a new human being’ rather than an individual and degrades bodily integrity [10]. Just as it is a major bodily invasion to force women to undergo an unwanted caesarean to preserve a foetus [21], it is a major bodily invasion to prevent women choosing MRCS because of potential, unquantifiable risks to a foetus, and/or on the basis of risks to her health that she is able to assess for herself.

Amu et al. argue that restricting MRCS decisions is justified because MRCS is often irrational [3]. Decisions about which risks in childbirth to assume is a highly personal choice, which can be based on any number of personal attributes, cultural or social factors [53]. There are rational reasons women may want to assume some risks over others, or assume some risks to get certain reassurances [32]. An individual’s perceptions of significant risks are important in medicine [15] and this is not negated by pregnancy. Further, in examining a woman’s reasons we are assessing decision-making by the standards of reasoning deployed [65], rather than their capacity to make the decision. It is discriminatory to project a more stringent standard of decision-making onto pregnant women. Jackson argues that judging a woman’s reasons for seeking a termination is inconsistent with common law principles of self-determination [28]. Her argument equally applies to examining a woman’s reasons for choosing one form of childbirth over any other. Examining reasons singles pregnant women out as a group to be ‘managed’ by extraordinary measures. Moreover, it fails to recognise the importance of subjective experience, highly valued in law [39], in assessing risk. Competent women are, and should be, entitled to make decisions about childbirth, even if it appears irrational to obstetricians. Some authors contend, however, that pregnant women are unable to make autonomous choices about childbirth [11].

Autonomy is more than just a matter of access to options [12]. Choices are only sufficiently autonomous when made with understanding [66], thus reflecting true wishes. Informed choice is exercised when patients freely and intentionally choose a medical procedure, over another, ‘after having substantial understanding of the proposed medical treatment[s]’ [66]. Some claim that because MRCS is elective, and evidence is complex, it is difficult for women to make MRCS decisions. To make a sufficiently autonomous decision women must understand that they are potentially assuming risks that may otherwise not be present [11]. This understanding, and other information, can be obtained with good counselling about childbirth choices. Two objections are usually made to this claim. First, sufficient counselling (and therefore sufficient understanding) is impossible because there is often not time to discuss options in detail. Burcher et al. [11] posit that women often ask for MRCS once already in labour. This is an extreme (and narrow) example. It does not demonstrate anything inherent to MRCS that women cannot understand, only that time must be spent earlier in pregnancy ensuring women understand their options. Wagner argues that even when there is time for discussion, women are not ‘patient enough’ for thorough discussions of risk [62] necessary for fully formed decisions. Supporters of MRCS do not have to defend instances where doctors or patients do not take their obligation to explain and listen seriously. Patients never have as thorough an understanding of risks as doctors, but that does not mean they lack sufficient understanding to consent. There is willingness, and patience, amongst women seeking MRCS to understand the risks. Birthrights indicate that most women seeking MRCS conduct their own extensive research into the available evidence [9]. Second, some claim that women, even with proper counselling, cannot exercise autonomy about childbirth fully because fear interferes with decision-making [11]. Fear of childbirth can also be rational because childbirth is always risky and painful. Moreover, fear is common to most medicalised processes, but we do not often treat patients in circumstances outside of pregnancy with the same suspicion. Fear does not inherently preclude understanding. There might be cases when a woman cannot weigh risks effectively because fear becomes *paralysing*,^19^ but these individual instances should not be used to generalise. It must be assumed that most women are as competent to decide their childbirth, as they are their cancer treatment. It is also inaccurate to paint MRCS as solely motivated by fear [9]. MRCS is most often the result of individual women’s engagement with evidence and their circumstances [9].

Maximally autonomous decisions are those choices free from interferences, such as disproportionate social pressure [13]. Burrow expresses concern that the over-medicalisation of childbirth subjects women to overwhelming pressure from doctors and society to conform to certain perceptions of risk [13]. Burrow seeks to enhance women’s agency, but her arguments about women being subject to undue pressure can be utilised to deny choice as much as they can encourage nuanced discussion about empowering women against certain conditions. There may be social pressure to elect for MRCS because it is more ‘desirable’ [13], to prevent vaginal injury (from delivery) and resultant ‘interference with sexuality’ [4]. This pressure can unwittingly influence women’s decisions, Burrow argues, because critically reflecting on social influence is difficult [13]. The potential for autonomy to be undermined should not be used as grounds to deny MRCS as a live option. Rather it highlights that effective counsel is crucial to minimise the impact of social pressures. MRCS does not inherently control or perpetuate narratives about childbirth even if it may sometimes be a response to them. Liberal societies do not limit choice because of harmful social narratives that can surround choices without proof the choice is inherently harmful. Instead, we deal directly with the narrative. Narratives about the vagina and how the vagina must be for a partner’s sexual pleasure are potentially already harmful to women. Using these harmful narratives to deny women, who genuinely want MRCS, choice only compounds the harm caused. Moreover, how can we know which narratives are patriarchal and which speak to womens' wellbeing and individual concerns about bodies? Assuming that all concern about future sexual experiences after childbirth are *only* about men is hetero-normative, patronising and fails to recognise that women care about their future bodies for their own reasons. Effective counselling is the best opportunity to discuss some of the pervasive and harmful myths about childbirth that could permeate in social narrative whilst recognising women’s genuine concerns. Committing to liberalism and respecting individual liberty would mean allowing women freedom to make a choice in childbirth, despite these external influences. The provision of counselling would ensure that the freedom to make a choice is also autonomy enhancing because it facilitates intentionality, understanding and non-control. Finally, completely uninfluenced decisions are impossible. If persons were only empowered to make choices *entirely* absent any societal influence the range of decisions anyone could make would be incredibly limited. It would be absurd to use potential influence to deny women agency about childbirth, especially given its lasting impact on the body.

### The MRCS Risk Fallacy

Lots of studies demonstrate caesareans have higher mortality rates,^20^ harbour greater risk of hysterectomy [52] and respiratory morbidity in infants [36]. Following caesarean women are more likely to experience miscarriage or stillbirth in future pregnancies [46]. The evidence about *caesareans* appears definitive, however it does not establish that *MRCS* is significantly risky. On closer inspection evidence about the riskiness of MRCS risk is more inconclusive. Studies about caesarean risk use data from pregnant women who needed emergency caesareans for medical reasons [23]. It is difficult to extricate dangers inherent to caesarean from the underlying medical emergency [23]. When a woman’s circumstances are already dangerous the outcomes of a caesarean will likely have worse outcomes. Caesarean outcomes may be much better [46] when there are no underlying medical complications. Bewley and Cockburn posit that some adverse outcomes associated with caesareans are not accounted for by emergency conditions [7]. Notably they provide no examples of a complication that is not compounded by circumstances. Even the 1.9% chance of a surgeon’s scalpel causing foetal injury [32] is impacted by emergency circumstances because of pressurised conditions experienced by the surgeon or increased distress/movement in the pregnant woman. Wagner argues there is correlation between MRCS and maternal mortality. He suggests that data in the UK recording deaths in childbirth^21^ shows a 2.84 relative risk of death in non-emergency caesareans [62]. This data, however, only demonstrates that there are some instances in which non-emergency caesarean results in death: it does not inform about the likelihood of death. It remains unknown how many women opt for MRCS without dying. Further, his observations, not being the result of a controlled study, do not account for a multitude of confounding variables impacting on MRCS outcomes. No reliable data exists about the risks of MRCS [3]. It is reasonable, however, to assume that in the absence of emergencies it is easier for surgeons to implement effective safety protocol and carefully manage the procedure. Keag et al. [31] conclude that MRCS, when the short-term effects of caesarean are minimised, could be as safe as VD. Even if there is additional risk in MRCS the outcome of MRCS is likely to be better than necessary (emergency) caesareans so we should not rely on caesarean data to restrict MRCS access.

Sometimes the risks associated with *caesareans* are presented unfairly to make MRCS seem irrational. Authors often cite a greater relative risk of death in MRCS [62]. This relative risk, however, does not establish the likelihood of mortality in any given case [45]. Even if MRCS is twice as likely to result in death, that risk is much less significant if a 1% chance of death in childbirth increases to 2%, rather than a 10% chance becoming 20%. Discussion of relative risk without context seems a deliberate tool to make MRCS seem more dangerous. Moreover, the risks of *caesareans* are rarely discussed comparatively. No pregnancy, irrespective of delivery, is without risk to life.^22^ Risks of VD include pelvic floor injury [60], and increased likelihood of stillbirth [45] or incontinence [36]. Women who opt for VD often receive some technological assistance (forceps or vacuum). Assisted delivery, compared to spontaneous delivery, carries increased risk of haemorrhage or wound complications [63]. 25% of assisted deliveries result in long-term incontinence [45]. Whilst MRCS potentially increases some risk, it does avoid some of inherent risks of VD [44], such as incontinence,^23^ and pelvic floor injury.^24^ Caesareans also minimises risk to foetus of prolonged pregnancy or birth injury [44]. MRCS decisions are broader than just the risks and benefits of elective caesarean. Decisions should involve comparative consideration of the risks of other childbirths.

Presumed increase of maternal morbidity and mortality in MRCS is the most common justification given to restrict MRCS, but increase in morbidity is not clearly evident [43]. Uncertainty is significant [67]. For some, clinical uncertainty about the risks of MRCS compared to VD [23], means there are grounds [23] to allow women choice. For others, uncertainty alongside knowledge that emergency caesareans are riskier is sufficient justification for preventing choice. Others posit that uncertainty means MRCS should not be refused, but should not be routinely *offered* to patients [43]. It is clear more research is needed [23], and is supported by obstetricians [64],^25^ to resolve uncertainty. Wax et al. [63] believe a thorough study, comparing MRCS and spontaneous delivery, will resolve the risk issue, and thus the MRCS debate.^26^ To some extent, it is clear that doctor’s willingness to provide MRCS is dependent on clinical evidence, rather than women’s opinions about what risks they want to assume in childbirth (absent an emergency). This is indicative of the limited trust placed on women to exercise they autonomy in pregnancy, which I have already established should not translate into a reduction of women’s choices.

## Non-maleficence: Absence (?) of Clinical Need

MRCS, it is sometimes claimed, is ethically impermissible because it is a surgical procedure that, in the absence of clinical need, causes unnecessary harm. The principle of non-maleficence (do no harm) sometimes justifies interference with even autonomous choices [11] potentially including MRCS. While a key argument against MRCS is the absence of clinical need, some doctors believe there are clear, and common, clinical indications for elective caesarean [60] such as preventing pelvic floor disorders [43]. There is some inherent bias in determining what *clinical need* [20] encompasses. Arguments reliant on the ‘absence of clinical need’ often generalise about women’s experiences. Women do not all have the same physical experience of pregnancy, experience the same risks or place equal weight on those risks. ‘Clinical need’ is overly based on traditional ideas about medicine as a response to pathologies. The majority of women contacting Birthrights about MRCS had previous experience of traumatic birth, concerns about an underlying health condition falling below the threshold of emergency, primary tokophobia, or sexual assault [9]. Many of these reasons easily fit within ‘clinical need’ [20] if a holistic approach is taken to physical and mental health. The inherent bias in classifying ‘clinical need’ (or absence of) manifests in doctors' assessments of the reasons for MRCS. For instance, some doctors believe tokophobia is sufficient to demonstrate need for caesarean as a measure preserving mental health [7]. Others believe caesarean is not primary treatment [7], and MRCS legitimises tokophobia as a biomedical diagnoses, treated invasively, when it is iatrogenic [22]. Support must be provided to women with tokophobia [7], but that does not mean MRCS is always an inappropriate management option. Denying the experiences of women could result in significant psychological trauma. This is also true for women with other experiences beyond tokophobia. For example, women who place significant value on minimising other risks like incontinence, for example, because of underlying digestive disorder. Broader views of welfare in clinical decision-making^27^ ensure that doctors best protect individual interests, and physical and mental wellbeing.

Women do also sometimes seek MRCS for reasons of convenience, or because their last VD was a negative experience, or because of perceived negative effects of VD on their sexual function and relationships [6]. These factors are unlikely to be incorporated within, even a broad approach to, clinical need. Bewley and Cockburn posit that doctors should be concerned with pathology rather than fashions within physiology [7]. They explain that pregnancy and childbirth are normal physiological processes [7] rather than illness [62]. MRCS is, thus, an unnecessary, ‘unnatural’ intervention within a natural process. Wagner compares MRCS to the patient demanding antibiotics for a viral infection. He argues that just as the responsible doctor refuses to administer antibiotics when they would not treat the ailment, responsible doctors refuse MRCS because it treats no ailment [62]. VD is a normal physiological process. Most pregnant women deliver vaginally^28^ and wish to do so [13]. It is still, however, a difficult experience. All medical supervision and practice is the intervention of the ‘unnatural’ [45], but medicine is not, and should not, be wholly reserved for pathologies. There are plenty of examples where it is considered medically appropriate to use surgical interventions in the absence of pathology. For example, gastric-band surgeries are surgical interventions that attempt to achieve something (weight loss) that could be otherwise be achieved naturally. Moreover, medicine has the potential to alleviate some of the difficulties in pregnancy and women are entitled to utilise that potential for relief. Lack of pathology was used by doctors to resist the introduction of analgesics in the management of labour in the nineteenth century,^29^ but the benefits of providing painkillers are now well established. It would be cruel and regressive to deny women the option of epidural to relieve pain during labour. Normal pregnancy progression is, thus, not an obvious barrier to MRCS if a woman believes it is an important form of assistance.

Wagner argues that women know VD is the normal and inevitable physiological consequence of carrying a pregnancy. They are, thus, responsible for VD when deciding to carry a pregnancy [62]. His argument is that pregnant women may be entitled to analgesics and supervision in childbirth, but not an alternative to childbirth altogether. He contends that denying women MRCS is not the same as forcing a VD, because this is a fact of pregnancy. This ignores obstetrical realities and the options that women could, if allowed, access. Caesarean is increasingly routine, and in the public consciousness. Forcing women to follow the course of a normal physiology, when there are alternatives is an unreasonable physical burden to place on women. MRCS is not unjustified or unethical just because it is unnatural. Wagner’s position is reminiscent of historical narratives condemning women to their ‘biblical sentence to painful childbirth’ [57].

MRCS does assume some risks different in nature to those in VD. The surgery causes damage to the woman’s body that otherwise could not have happened. This is true of all surgeries, but the argument is that in the absence of necessity it becomes actively harmful [7] because it is avoidable. There is no guarantee that the outcome of VD is non-harmful (for a woman’s physical and mental well-being), but objectors to MRCS argue that the principle of non-maleficence only justifies surgical delivery when there is significant risk VD would cause more harm. Non-maleficence is, however, not always the guiding principle in determining the permissibility of a procedure. We should not neglect the ‘request’ element of MRCS when analysing harm. Significant harm is caused to individuals when doctors ignore their requests in place of clinical judgement. This is of particular significance in the context of childbirth, for reasons I have already explored, and of greater importance than notions of *unnecessary harm*.

## Justice: Inequitable Cost (?) of Choice

There is a difference between acknowledging that MRCS is permissible and so ought to be available, and arguing that it must be routinely available. It is often argued that even if ethically permissible, demands of justice mean MRCS should not be publically funded. Yamamoto posits that ‘in the spirit of… equal distribution of care it is difficult to justify… [MRCS] as an alternative to the traditional mode of delivery’ [67]. Wax et al. [63] argue that because MRCS involves a patient choosing one more expensive treatment over other assistance in the absence of necessity, the patient should be responsible for the additional associated costs of their choice. These cost concerns are not reason to disallow MRCS,^30^ but are claims that women who choose MRCS should be responsible for additional costs to prevent their choice becoming an unfair burden on others. Arguments restricting publicly funded provision of MRCS only work if MRCS is of *sufficient* additional cost than VD.

Additional costs associated with caesareans come from the additional resources needed to perform preoperative checks, the procedure [34] (including anaesthetic, antibiotics, equipment, surgical time etc.), and post-operative hospital stays (usually estimated to be twice as long) [46]. Some estimates place the cost of providing MRCS at around £15million [22]. The evidence of significant additional cost, however, is not so clear-cut. £15million may sound expensive, but may not be so consequential in the context of a public health system’s budget.^31^ Moreover, Miensk and Reale argue that ‘uncomplicated’ and well-managed MRCS may differ little from, or may be less costly than, a technologically managed VD [36]. Significant resources are also deployed during a managed VD, such as analgesics, antibiotics, surgical equipment and medical supervision. In the medicalised model of supervised childbirth these costs are frequently incurred. NICE estimate the cost difference between a well-managed MRCS and medically supervised VD is only £84 [9]. This estimate could perhaps be criticised for neglecting differences in aftercare, but there are often significant inconsistencies in necessary aftercare following any birth depending on individual circumstances. It is, thus, difficult to fairly estimate a difference. NICE concluded that the additional cost of MRCS was not significant enough to prevent MRCS being provided with public funds [9]. Furthermore, the substantial difference in cost between funding and not funding MRCS depends entirely on the number of women choosing MRCS. Cost is limited if only a small percentage of women seek MRCS. The majority of women choose VD, even after MRCS counselling [20].^32^ The cost trade-off in any event is minimal. It is worth incurring the (likely substantial) cost of analgesics to minimise distress in childbirth. It is equally worth incurring some minimal additional cost to provide MRCS to minimise distress.

Justice also requires us to consider access. The importance of choice in childbirth means it should not be determined by lottery. NICE guidelines intended to achieve standardisation, but have failed because of the inconsistency in their interpretation and application. Limiting MRCS only to private patients would further compound the existing lottery problem. Lottery is unfair because of the significance of birth as an event in a pregnant woman’s life, and the degree to which women are having their wishes ignored. Ignoring childbirth choices has traumatic and difficult consequences for women. It is concerning that Trusts and individual doctors are not consistent in their approach to how much choice they offer pregnant women about childbirth. Denying women, and at random, this choice is an affront to their autonomy and bodily autonomy. Stricter adherence to NICE guidelines, and therefore greater accessibility of MRCS, is an ethical imperative.

## Conclusion

This paper has demonstrated that women are being consistently denied choice in childbirth because individual doctors and pragmatic realities are the actual determinants of access to MRCS. NHS Trusts and CCGs have failed to implement, or consistently implement, NICE guidelines. The routine denial of choice, without consistent justification, is a significant limiting factor on pregnant women’s autonomy. In this paper I demonstrated that interfering with women’s decisions about MRCS is not defensible. By examining the key ethical principles of autonomy, beneficence, non-maleficence and justice I argued that access to MRCS should be protected to allow women to make choices in childbirth based on subjective preferences and experiences. It is clear MRCS is a ‘reasonable alternative’ [39] to VD because it is not clinically unjustifiable [44]. There is insufficient evidence to suggest MRCS carries significant additional risks compared to VD that women are incapable of understanding and using to make decisions themselves. Doctors are perhaps already, and certainly should be, under an obligation to discuss MRCS as an option with patients. Routine counselling, that is both neutral and thorough, about *all* choices in childbirth, including MRCS, is the only way to ensure *all* women understand the options in childbirth and can make any choice. NHS Trusts have not done enough to ensure access to choice. Moreover, NICE guidelines, in stating that MRCS should not be routinely discussed and allowing conscientious objection, have entrenched the lottery, and thus do not go far enough to meet their stated aim of making women the primary decision makers in childbirth [40]. The focus of future discussion, therefore, should be how we can reduce this lottery and ensure access to choice.

## Footnotes

1 A caesarean section is the surgical delivery of a foetus through an incision into the abdomen and womb of a pregnant woman.

2 Such as anesthetics, antiseptic practices and antibiotics.

3 It is suggested that at least some of the caesarean births in the UK are maternal request because experts estimate that in a birthing population surgical intervention will be necessary in around 19% of births. See: Molina et al. [38]. In July and August 2018 it was estimated that 14% of all births in England were by caesarean. See: NHS Digital (2018). Maternity Services Monthly Statistics. https://digital.nhs.uk/data-and-information/publications/statistical/maternity-services-monthly-statistics. Accessed 17 December 2018.

4 Tokophobia is the fear of childbirth.

5 The NICE Charter states that while ‘health and social care professionals are actively encouraged to follow our recommendations to help them delivery the highest quality care… our recommendations are not intended to replace the professional expertise and clinical judgement of health professionals, as they discuss treatment options with their patients.’ See: National Institute for Health and Care Excellence (2017). NICE Charter 2017. https://www.nice.org.uk/Media/Default/About/Who-we-are/NICE_Charter.pdf. Accessed 29 October 2018. It is also possible for Trusts and CCGs to depart from guidance when they can give good and detailed reasons for departing from guidelines: *R (on the application of Elizabeth Rose) v Thanet Clinical Commissioning Group* [2014] EWHC 1182 (Admin).

6 For example, doctors are entitled to object, on the grounds of conscience, to provide pregnancy terminations when the termination is not necessary to save the life of the pregnant woman. See: Abortion Act [1], s.4.

7 This right is contained in the NICE guidance about MRCS, and also contained in the general principle that a doctor cannot be compelled to perform a medical intervention for a patient if there is no emergency following *R (Burke) v General Medical Council* [49].

8 The percentage of doctors that are accepting of MRCS in the UK is one of the highest in Europe. See: Habiba et al. [23].

9 It is estimated that around 33% of UK Obstetricians object to abortion, though this does not necessarily establish that these doctors would refuse to provide terminations to patients under the Abortion Act 1967. There is reason to believe that the number of conscientious objections is increasing, and may continue to increase. See: International Women’s Health Coalition (2018). Unconscionable: When Providers Deny Abortion Care. https://iwhc.org/wp-content/uploads/2018/06/IWHC_CO_Report-Web_single_pg.pdf. Accessed 29 October 2018.

10 There are arguments as to whether contentious objection ever appropriate at all in medical practice, but I do not wish to delve into this broader point in this paper, and comment instead only in relation to conscientious objection and childbirth.

11 This is discussed in more detail later in this paper.

12 Emily Jackson makes this argument in the context of abortion decisions in Jackson [28]. The law is clear that a competent adult is entitled to make whatever decision about medical treatment they wish even if their reasons are irrational: Re [51].

13 There was a tendency in the literature for some writers to be more critical of decisions based on convenience, for example, rather than instances where women make decisions based on the avoidance of certain risks.

14 NHS Digital publishes a monthly report that details delivery methods. Emergency and elective caesareans are recorded separately. The data may not be reliable for several reasons. The data is based on Trusts self-reporting. Data is only provided by Trusts in England, and some Trusts do not regularly supply data. There is also no information contained in the report about how Trusts classify caesareans as ‘emergency’ or ‘elective’.

15 In law refusal to respect patient’s autonomous preferences is generally limited to those individuals who do not have the capacity to make decisions.

16 The law no longer supports wholesale paternalistic decision-making in medicine. The law affords the highest priority to the patient’s right to autonomy. Lord Steyn commented that ‘in modern law, paternalism no longer rules’ in *Chester v Afshar* [15] UKHL 4. This, of course, might not rule out the possibility of therapeutic privilege being deemed permissible in tightly determined circumstances. In fact *Montgomery v Lanarkshire Health Board* [39] UKSC 11 left open the possibility that a doctor may have a complete defence to negligence in instances of withholding certain risks from a procedure prior to treatment, where it can be established that there are therapeutic reasons for an exception from the ‘duty to disclose.’ This was noted in the Court of Appeal in *Chinnock (Schumann) v Veale Wasbrough* [2015] EWCA Civ 441 [60] per Jackson LJ. Mulheron observes, however, that there are few cases where therapeutic privilege has been expressly endorsed by judges and only one clear instance where therapeutic privilege was able to preclude a doctor’s liability. See: Mulheron, R., (2017) Has Montgomery Administered the Last Rites to Therapeutic Privilege? A Diagnosis and a Prognosis. *Current Legal* Problems 70(1), 149–188. Therapeutic privilege would rarely be deemed appropriate in law, and in this paper I argue why it is insufficient justification to prevent women choosing between MRCS and VD.

17 Note that this argument must assume that there is access to abortion.

18 The risks are uncertain and risks in relation to the foetus are inconclusive. Studies do suggest increased incidence of respiratory distress in neonates after MRCS (Plante [46]), however evidence also suggests that this is the result of caesareans being undertaken before the end of the complete gestational period. Planning caesareans as close to the 39 weeks as possible largely mitigates the risk (Paterson-Brown [44]). Moreover, there may be instances in which planned elective caesarean, whilst not medically necessitated, may still reduce some risks to the foetus: such as birth injury or stillbirth (Penna [45]).

19 The argument that severe fear precluded sufficiently autonomous decision making during childbirth was accepted in *Re MB (Medical Treatment)* [50] 2 FLR 426, in which a patient agreed to a caesarean section but refused anaesthetic because of a needle phobia, making the caesarean impossible. The judges held that MB lacked the capacity to refuse the anaesthetic because her phobia of needles ‘dominated her thinking and made her quite unable to consider anything else.’ Legal commentators have praised this decision for protecting women from making insufficiently autonomous choices. See: Herring [26].

20 Some studies place the relative risk of death from caesarean at 3.8 (Schuitemaker et al. [54]) and others at 4.5 (Hall [24]).

21 Wagner cites interpreted data from the UK Confidential Enquiries into Maternal Deaths. See: Hall & Bewley [25].

22 Even in developed countries. See: Maternal Mortality: How Many Women Die in Childbirth in Your Country? The Guardian. https://www.theguardian.com/news/datablog/2010/apr/12/maternal-mortality-rates-millennium-development-goals. Accessed 19 March 2018.

23 In caesareans the risk of incontinence, though prevalent, is much reduced. See: Nygaard and Cruikshank [43].

24 Having a caesarean virtually eradicates the risk of pelvic floor injury. See: Nygaard and Cruikshank [43].

25 In a survey of obstetricians in Maine 82.1% respondents felt it was important to hold, and fund, a trial of the outcomes of elective caesareans vs. spontaneous vaginal delivery in order to ascertain a better understanding of the risks. They felt that this would be instructional in resolving the MRCS debate. See: Wax et al. [64].

26 Plante posits that in order to get reliable data there would need to be a large and randomised control trial to limit the impact of other variables on the outcome of childbirth. Such a trial is, however, unlikely because an insufficient number of women, and doctors, would be willing to cede control over childbirth to random allocation (Plante [46]). It seems unlikely there will ever be a comprehensive comparison of the risks in MRCS and vaginal delivery.

27 A more holistic approach to welfare is particularly evident in cases concerning patients who lack capacity and difficult treatment decisions. The law is clear that when considering the best course of action the patient’s best interests must be considered. This requires consideration of issues broader than only their medical interests, such as emotional and social issues. See: *Re MB* (*Medical Treatment*) [50] 2 FLR 426; Re A (Male Sterilisation) [2000] 1 FCR 193.

28 Only 29% of births in the United Kingdom are surgical. See: NHS Digital [42].

29 Famously clergy in the nineteenth century claimed that ‘Chloroform is a decoy of Satan, apparently offering to bless women, but in the end it will poison society and rob God of the deep earnest cries which arise in time of trouble for help!’ See: Kurapati [33].

30 If women do not use additional communal resources to give effect to their MRCS decision there is no harm on which to justify the restriction of MRCS.

31 Planned NHS budget for 2017/2018 is estimated to be £124.7 billion. See: The King’s Fund (2018). The NHS budget and how it has changed. https://www.kingsfund.org.uk/projects/nhs-in-a-nutshell/nhs-budget. Accessed 10 October 2018.

32 In Italy, where the law requires that women’s choices in childbirth including MRCS be respected, only 4% of women opt for MRCS. See: Paterson-Brown [44].
